# Effort-Reward Imbalance Is Associated With Alcohol-Related Problems. WIRUS-Screening Study

**DOI:** 10.3389/fpsyg.2019.02079

**Published:** 2019-09-13

**Authors:** Jens Christoffer Skogen, Mikkel Magnus Thørrisen, Tore Bonsaksen, Jussi Vahtera, Børge Sivertsen, Randi Wågø Aas

**Affiliations:** ^1^Department of Health Promotion, Norwegian Institute of Public Health, Bergen, Norway; ^2^Alcohol and Drug Research Western Norway, Stavanger University Hospital, Stavanger, Norway; ^3^Department of Public Health, Faculty of Health Sciences, University of Stavanger, Stavanger, Norway; ^4^Department of Occupational Therapy, Prosthetics and Orthotics, Faculty of Health Sciences, Oslo Metropolitan University, Oslo, Norway; ^5^Faculty of Health Studies, VID Specialized University, Sandnes, Norway; ^6^Department of Public Health, University of Turku and Turku University Hospital, Turku, Finland; ^7^Department of Research and Innovation, Helse Fonna HF, Haugesund, Norway; ^8^Department of Mental Health, Norwegian University of Science and Technology, Trondheim, Norway

**Keywords:** alcohol, psychosocial working-environment, effort-reward imbalance, alcohol-related problems, work, health-related behaviors, lifestyle

## Abstract

There is ample evidence of associations between a perceived stressful working environment and several health-related outcomes. To better understand potential mechanisms behind these observations some studies have focused on the relationship between effort-reward imbalance at work and alcohol consumption. So far, the findings have been inconsistent. One reason for this inconsistency might come from the focus on alcohol consumption *per se*, while disregarding other aspects such as adverse consequences related to the consumption of alcohol. The aim of the present study was to explore associations between perceived effort and reward, effort-reward imbalance and overcommitment, and alcohol-related problems. Using data from the alcohol screening component in the Norwegian WIRUS-project (*N* = 5,080), we ascertained the perceived effort, reward, effort-reward imbalance (ERI) and overcommitment using the effort-reward imbalance questionnaire. Alcohol-related problems was determined using a cut-off ≥8 on the Alcohol Use Disorder Identification Test (AUDIT). Associations were estimated using crude and adjusted logistic regression models. Covariates were age, gender and education. We found associations between different aspects of ERI and overcommitment, and alcohol-related problems. Specifically, the main analysis indicated that there was an increased odds for alcohol-related problems among those who reported high levels of ERI in conjunction with high overcommitment [adjusted OR: 1.40 (CI 95% 1.10–1.78)] compared to those with low levels of ERI and low overcommitment. Our findings suggest that ERI and overcommitment is associated with increased likelihood of alcohol-related problems. These findings indicate that individual and work-related factors should be taken into account collectively when aiming to determine the impact of psychosocial work environment on alcohol-related problems. Due to the cross-sectional nature of the present study, we are not able to determine the direction of the associations, and future studies should aim to investigate this.

## Introduction

There is ample evidence of an association between a perceived stressful working environment and several health-related outcomes, including cardiovascular disease (Kivimäki and Kawachi, 2015; Pikhart and Pikhartova, 2015), cancer (Eddy et al., 2016), immune functioning (Eddy et al., 2016), sleep quality (Van Laethem et al., 2013), mental health problems (Stansfeld and Candy, 2006; Finne et al., 2014; Einarsen and Nielsen, 2015; Theorell et al., 2015; Harvey et al., 2017), musculoskeletal disorders and pain disorders (da Costa and Vieira, 2010; Bernal et al., 2015; Herr et al., 2015; Madsen et al., 2018). Moreover, work-related stress has been shown to inflict substantial financial cost to society (Hassard et al., 2018). In an effort to understand the underpinnings of the observed associations, some studies have focused on the relationship between the psychosocial working environment, its interaction with the employees, and specific health-related behaviors, (Stansfeld and Candy, 2006; Heikkila et al., 2013; Kivimaki et al., 2015; Kivimäki and Kawachi, 2015; Sara et al., 2018) such as alcohol consumption, heavy drinking, and alcohol misuse (Puls et al., 1998; Head et al., 2004; Kouvonen et al., 2005; Guo et al., 2006; Marchand, 2008; Fear et al., 2009; Azagba and Sharaf, 2011; Marchand et al., 2011; Barnes and Zimmerman, 2013; Van Rafelghem et al., 2018; Medisauskaite and Kamau, 2019).

One of the largest studies to date is a meta-analysis of individual-level data by Heikkila et al. (2013) from several different cohorts in Europe with 140,000 participants (Heikkila et al., 2013). The authors considered the cross-sectional and longitudinal association between experienced job-strain, a measure of work stress [according to Karasek’s Demand-Control model, as measured by the Job Content Questionnaire (JCQ; Karasek et al., 1998)] and alcohol consumption. In the cross-sectional data, they found slightly higher odds of job-strain among non-drinkers and heavy drinkers and slightly lower odds among intermediate drinkers compared to moderate drinkers, but longitudinal analyses did not support any clear associations between job-strain and alcohol intake. On the other hand, a systematic review and meta-analysis of over 330,000 participants across 61 studies, found that individuals whose working hours exceed standard recommendations were more likely to increase their alcohol use to levels that constitute a health risk (Virtanen et al., 2015).

The lack of congruence between these findings warrants further investigation into the influence of perceived stressful working environment on alcohol habits. One such factor may be when employees experience a lack of fairness, or imbalance, of the reciprocity of efforts expended and rewards received at work (Siegrist, 1996). This has been termed effort-reward imbalance (ERI), and have been found to be associated with poor health and adverse health-related behaviors (Ostry et al., 2003; van Vegchel et al., 2005; Rydstedt et al., 2007; Stanhope, 2017). The ERI-model is a framework concerned with how perceived poor reciprocity between job efforts and job rewards, such as salaries, promotion prospects, and job stability, constitute a detrimental factor that may result in adverse health outcomes. The model also posits that overcommitment, a coping strategy for employees striving for approval at work may further increase risk (Siegrist, 2017). In line with the ERI model, we would expect alcohol-related problems to be associated with high efforts, low rewards, a mismatch between efforts and rewards (effort-reward imbalance), and higher levels of work overcommitment. Only a few, mostly cross-sectional studies have examined the association of effort-reward imbalance with alcohol consumption (Puls et al., 1998; Head et al., 2004; Kouvonen et al., 2005; Guo et al., 2006; Jachens et al., 2016), and the results are mixed. For example, a large-scale study of over 40,000 public sector employees found no consistent association between ERI and heavy drinking (i.e., weekly consumption of 190 g or more absolute alcohol for women and more than 275 g for men) (Kouvonen et al., 2005). However, overcommitment was not included and ERI was assessed by a proxy measure in this particular study. Another study with a specific focus on humanitarian aid workers, only found support for a relationship between ERI and heavy drinking among females (Jachens et al., 2016).

The previously reported weak or inconsistent associations between job-strain, and effort and reward and alcohol-related problems are somewhat surprising and needs further scrutiny (Kouvonen et al., 2005). This is emphasized by previous research that give reason to believe that individuals who work more than standard recommendations are at increased risk for alcohol-related problems (Virtanen et al., 2015). One reason for the previous findings may be the frequent focus on alcohol consumption *per se*, while neglecting other aspects associated with alcohol-related problems, such as harm to oneself and other. This identifies a need for studies which measures different aspects of alcohol-related problems, such as consumption, binge drinking, harmful use and dependence [see discussion in Selin (2006) for instance (Selin, 2006)]. Therefore, the aim of this study was to explore associations between perceived effort and reward, effort-reward imbalance, and overcommitment, and alcohol-related problems. In the present study we used a compound measure – the Alcohol Use Disorder Identification Test (AUDIT) to assess alcohol-related problems (Saunders et al., 1993; Babor et al., 2001).

## Materials and Methods

### Design

The present study was conducted within a cross-sectional design, as part of the ongoing Norwegian national WIRUS project (Workplace Interventions preventing Risky Use of alcohol and Sick leave). Data for the current study was obtained from the alcohol screening component in the WIRUS project. More details and other results from the WIRUS project are published elsewhere (Aas et al., 2017; Nordaune et al., 2017; Thørrisen et al., 2018, 2019a,b; Skogen et al., 2019).

### Study Population and Sample

Data from employees in 20 large companies in Norway were included. The recruitment procedure were developed by University of Stavanger. The companies were recruited by the Drug and Alcohol Competence Center, Rogaland in cooperation with three Occupational Health Services (OHS), accredited by the Norwegian Labor Inspection Authority. The companies operated within both private (*n* = 8) and public (*n* = 12) sector, and employed approximately 18,000 employees within the following work divisions [as categorized according to the European Classification of Economic Activities (Eurostat., 2008)]: Public administration (*n* = 8), human health and social work activities (*n* = 4), manufacturing (*n* = 4), transportation and storage (*n* = 1), education (*n* = 1), accommodation and food service activities (*n* = 1), and other service activities (*n* = 1).

Individual-level inclusion criteria included: (a) employed in a company served by one of the participating occupational health service units, regardless of work division or geographical region, (b) status as employee (blue, white or pink collar worker, or manager, i.e., salaried person), (c) aged 16 to 72 and (d) basic understanding of the Norwegian language.

Employees’ email addresses were provided by the companies, and employees (*n* = 18,000) received a web-based questionnaire, information about the study, and an invitation to participate. A total of 5,080 (28.2%) employees agreed to participate.

### Independent Variable: Effort-Reward Imbalance and Overcommitment

ERI was measured by the short form of the ERI Questionnaire (Lau, 2008; Siegrist et al., 2008; Stanhope, 2017). As such, ERI takes not only the work content into account, but also the work role in a social perspective, as well as the employee’s individual coping pattern and need for control. The short version consists of 16 items, measuring three aspects: effort (3 items; ordinal Cronbach’s α 0.80), reward (7 items; ordinal Cronbach’s α 0.69) and overcommitment (6 items; ordinal Cronbach’s α 0.86). For each of the sub-scales, the mean individual score was computed. The imbalance between effort and reward (ERI) was gaged according to recommended procedures (Sperlich et al., 2012; Stanhope, 2017). A ratio (ERIRATIO) of >1 was used to identify participants with ERI in favor of effort from those with no ERI or ERI in favor of reward (Stanhope, 2017). For overcommitment, we used a median split to distinguish between low and high levels.

### Dependent Variable: Alcohol Use (AUDIT)

A translated Norwegian version of the AUDIT was used in the present study, consisting of 10 items measuring different aspects of alcohol habits and potential negative consequences of these alcohol habits (Saunders et al., 1993; Babor et al., 2001). The original recommendation of a total score of ≥8 as an indication of alcohol-related problems (Babor et al., 2001) have in studies yielded favorable sensitivity and acceptable specificity (de Meneses-Gaya et al., 2009). Even though some studies have indicated that different thresholds should be applied for different groups (e.g., for males and females), a score of ≥8 has generally been accepted as an optimal cut-off for identifying alcohol-related problems (de Meneses-Gaya et al., 2009). A recent confirmatory factor analysis of AUDIT based on the WIRUS-study concluded that AUDIT consists of one factor, and that there were no indications of differences in the factor structure or metric across gender (Skogen et al., 2019). The recommended cut-off for potential alcohol-related problems of a total score of 8 was used in the present study, and a dichotomous variable was constructed.

### Covariates

Age, gender and educational level were included as potential confounders in this paper. Educational level was recorded as a four-level variable, discriminating between primary/lower secondary, upper secondary, university/college education up to four years and university/college education for more than four years.

### Statistical Analyses

**FIGURE 1:**
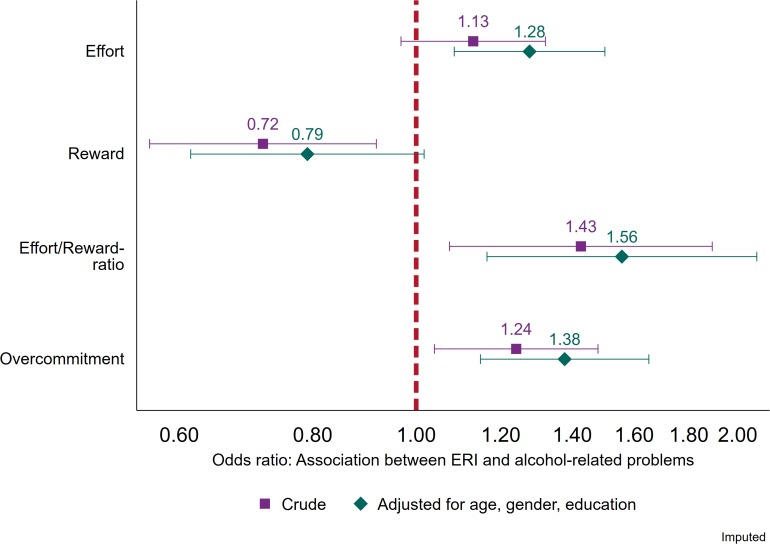
Associations between continuous measures of effort, reward, effort/reward-ratio and overcommitment, and alcohol-related problems (AUDIT ≥ 8). Logistic regression. Crude estimates, and estimates adjusted for age, gender, and education (*N* = 5,080).

First, the characteristics of those above and below the cut-off for potential alcohol-related problems across age, gender, educational attainment, and ERI-subscale mean scores were estimated and compared (Table 1). For age and the ERI-subscales, mean and standard deviations are presented, and independent samples *t*-tests were used to compare age and mean scores on subscales between the two AUDIT groups. For gender and education, proportions are presented, and χ^2^-tests were used to compare the gender and educational attainment between the two AUDIT groups. We also estimated the direction of association between primary relations of interest (alcohol-related problems and ERI-components), between covariates and exposure, and between covariates and outcome in the present study (Table 2). Next, separate logistic regression models were estimated for each of the ERI-subscales (effort, reward, and overcommitment) as well as for effort/reward-imbalance as independent continuous variables, and AUDIT as dependent variable (Figure 1). Crude associations as well as associations adjusted for age, gender and educational attainment were estimated for each of the sub-scales, and the point estimate change between crude an adjusted log odds ratio estimates were computed using the following formula:

$$\left(\left(\frac{A\,d\,j\,u\,s\,t\,e\,d\,\log o\,d\,d\,s\,r\,a\,t\,i\,o}{C\,r\,u\,d\,e\,\log o\,d\,d\,s\,r\,a\,t\,i\,o}\right)-1\right)\times 100.$$

**TABLE 1:** Characteristics of sample (*N* = 4,314).

	**AUDIT-score < 8 (*N* = 3,828)**	**AUDIT-score ≥ 8 (*N* = 486; 11.3%)**	***p*-value**
**Sociodemographics**			
Mean age (standard deviation; SD)	45.7 (11.1)	41.0 (12.6)	**<0.001**
Gender (% female)	69.0%	44.7%	**<0.001**
Education (%)			**=0.004**
Primary	2.3%	3.7%	
College/high-school	23.0%	28.4%	
University up to 4 years	33.9%	33.7%	
University 4+ years	40.8%	34.2%	
**Effort-reward imbalance**			
Effort mean (SD)	2.77 (0.61)	2.82 (0.59)	=0.15
Reward mean (SD)	2.69 (0.39)	2.64 (0.37)	**=0.01**
Overcomitment mean (SD)	2.29 (0.56)	2.35 (0.57)	**=0.02**

*Bold indicates significant differences at *p* < 0.05.*

**TABLE 2:** Direction of Association Between Primary Relations of Interest, Between Covariates and Exposure, and Between Covariates and Outcome in the Present Study.

	**Alcohol-related problems (outcome)**	**Gender (female)**	**Age**	**Education**
Alcohol-related problems		−	−	−
Effort (exposure)	+^*ns*^	+	+	+
Reward (exposure)	−	+^*ns*^	+^*ns*^	+
Overcommitment (exposure)	+	+	+^*ns*^	+
ERIRATIO (exposure)	+	+	−^*ns*^	+^*ns*^

*ns: *p*-value > 0.05.*

**FIGURE 2:**
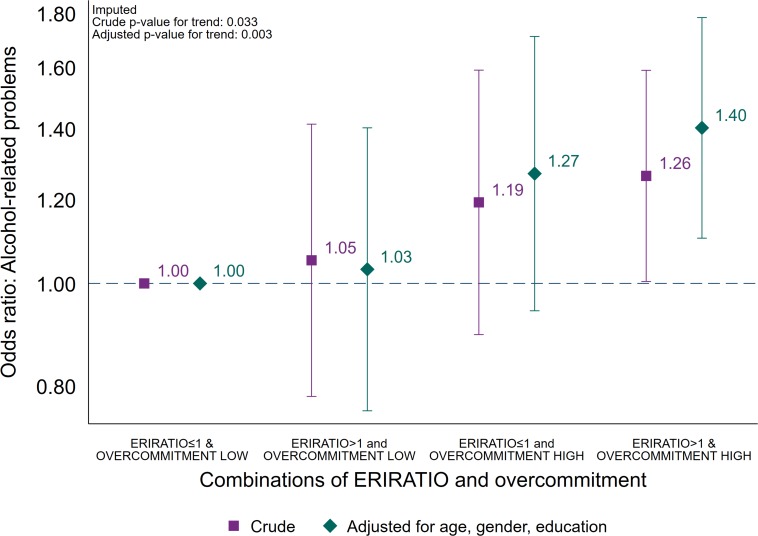
Association between combinations of effort/reward-ratio (≤1 or >1) and overcommitment (low or high), and alcohol-related problems (AUDIT ≥ 8). Logistic regression. Crude estimates, and estimates adjusted for age, gender and education (*N* = 5,080).

For the main analyses, a logistic regression model was computed using the following four combinations of effort/reward-imbalance and overcommitment: (i) ERIRATIO ≤ 1 and overcommitment low (reference group), (ii) ERIRATIO > 1 and overcommitment low, (iii) ERIRATIO ≤ 1 and overcommitment high, and (iv) ERIRATIO > 1 and overcommitment high as an independent variable (Figure 2). The main analysis was estimated as a crude model and as a model adjusted for age, gender and education, and again the point estimate change was estimated.

**FIGURE 3:**
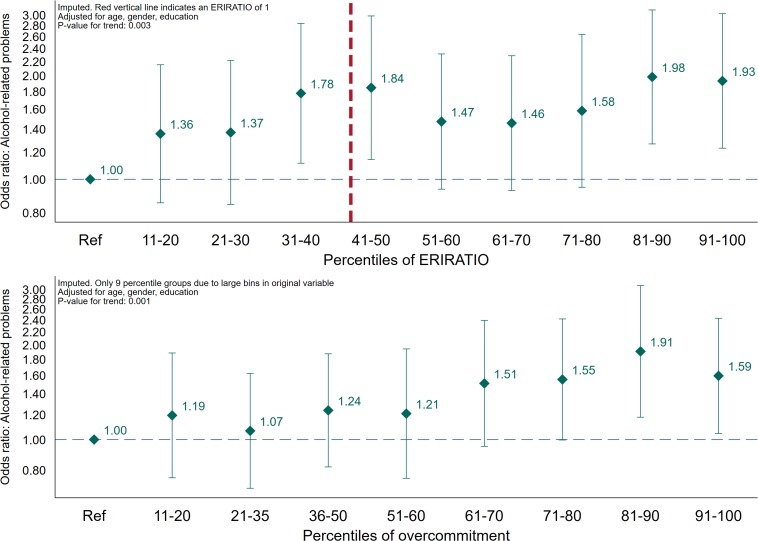
Association across percentiles of effort/reward-ratio and overcommitment, and alcohol-related problems (AUDIT ≥ 8). Logistic regression. Estimates adjusted for age, gender, and education (*N* = 5,080).

Finally, we estimated the association with alcohol-related problems across percentiles for ERIRATIO (deciles) and overcommitment (9 percentile groups due to large bins in original variable) in two separate logistic regression models adjusted for age, gender and education (Figure 3). Analyses were done using Stata version 15.1.

### Handling of Missing Information

Missing information ranged from *N* = 203 (3.8%) on gender and education to *N* = 966 (18.3%) on AUDIT. Those with missing information on AUDIT were younger (*p* = 0.004), more likely to be female (*p* < 0.001) and reported lower education (*p* < 0.001). With regards to the ERI-subscales, missing information on AUDIT was not associated with any of the sub-scales (*p* > 0.05). Logistic regression models in the main analyses was performed on multiple imputed datasets (Sterne et al., 2009). Twenty imputed datasets were created using the MI-procedure implemented in Stata. The results based on imputed datasets were similar to results from the non-imputed list-wise deleted dataset.

### Ethics Statement

Ethical approval for conducting the study was granted by the Regional Committee for Medical and Healthcare Research in Norway (no. 2014/647). The employees were informed about the study’s aim and confidentiality and were assured that participation was voluntary. All employees provided written informed consent to participate.

## Results

The main characteristics of the non-imputed sample is presented in Table 1. Among the eligible participants, 67.4% were female, and the mean age for the eligible participants was 45.0 (standard deviation 11.6) years. Individuals above the threshold (11.3%) for potential alcohol-related problems were somewhat younger (age 41.0 vs. 45.7), more likely to be male and report lower education compared to those below the threshold. Those above the threshold also reported less reward at work, and more overcommitment, while there was no difference in effort. For the main dependent variable, the group with low ERI (ERIRATIO ≤ 1) and low overcommitment constituted 33.0% of the non-imputed sample, while the two intermediate – *high ERI* (*ERIRATIO* > *1) and low overcommitment* and *low ERI (ERIRATIO* ≤ *1) and high overcommitment* constituted 16.7 and 15.6%, respectively. The group defined by high ERI (ERIRATIO > 1) and high overcommitment high constituted 34.6% of the sample. The direction of the association between primary relations of interest, between covariates and exposure, and between covariates and outcome are presented in Table 2.

The associations between the continuous ERI-subscales (effort, reward and overcommitment) as well as ERIRATIO (effort-reward imbalance ratio), and alcohol-related problems are presented in Figure 1. The effort sub-scale was not associated with alcohol-related problems in the crude (odds ratio (OR): 1.13; CI 95% 0.97–1.32) model but was significantly associated with an increased odds for alcohol-related problems after adjustments for sociodemographics (OR: 1.28; CI 95% 1.09–1.50; point estimate change + 98.9%). For the reward sub-scale, however, there was a decreased odds for alcohol-related problems in the crude model (OR: 0.72; CI 95% 0.56–0.92), but after adjustments for sociodemographics the association was rendered non-significant (OR: 0.79; CI 95% 0.62–1.02; point estimate change−29.0%). For continuous ERIRATIO, both crude (OR: 1.43; CI 95% 1.07–1.89) and adjusted (OR: 1.56; CI 95% 1.17–2.08; point estimate change + 24.9%) models were associated with increased risk for alcohol-related problems. This was also true for the overcommitment subscale (crude OR: 1.24 (CI 95% 1.04–1.48) and adjusted OR: 1.38 (CI 95% 1.15–1.65); point estimate change 48.5%).

For the main analyses, using the group defined by low ERI (ERIRATIO ≤ 1) and low overcommitment as reference, the two intermediate groups were not associated with increased odds for alcohol-related problems – high ERI (ERIRATIO > 1) and low overcommitment (adjusted OR: 1.03; CI 95% 0.76–1.40) and low ERI (ERIRATIO ≤ 1) and high overcommitment (adjusted OR: 1.27; CI 95% 0.94–1.71). The group defined by high ERI (ERIRATIO > 1) and high overcommitment high, however, significantly higher odds of alcohol-related problems (crude OR: 1.26 (CI 95% 1.00–1.59) and adjusted OR: 1.40 (CI 95% 1.10–1.78; point estimate change + 44.7%). The adjusted odds ratio for trend was 1.13 (CI 95% 1.04–1.22, *p*-value = 0.003) across each combination of ERI and overcommitment. Lastly, we found an increased risk of alcohol-related problems across percentiles of both ERIRATIO [trendOR: 1.05 (CI 95% 1.02–1.08, *p*-value = 0.003)] and overcommitment [trendOR1.07 (CI 95% 1.03–1.12, *p*-value = 0.001)] in logistic regression analyses adjusted for sociodemographics.

## Discussion

### Main Results

In the present study, we investigated the associations between aspects of effort-reward imbalance and potential alcohol-related problems. We found support for an association between ERI and overcommitment in relation to alcohol-related problems. Specifically, the main analysis indicated that there was an increased risk for alcohol-related problems among those who reported high levels of both effort-reward imbalance (ERRATIO > 1) and overcommiment (above median). Adjusting for age, gender and education increased the estimated association for this group with almost 50%, indicating negative confounding (“suppression”) of the included demographic variables. The continuous effort- and overcommitment-subscale, as well as ERIRATIO was associated with alcohol-related problems in the adjusted analyses, while the reward-subscale was not. Indications of negative confounding was also found for the continuous effort and overcommitment sub-scale, as well as for the continuous ERIRATIO, but not for the reward-subscale (positive confounding) in the analyses of the separate components of the ERI-scale. Finally, we observed an increased odds for reporting alcohol-related problems across percentiles of both ERIRATIO and overcommitment, but the pattern was more consistent for the latter. We did, however, not observe any particular increase in risk of alcohol-related problems at ERIRATIO-levels above 1, which has been suggested as the critical cut-point in the ERI-model (Siegrist, 2017).

In our sample, the negative confounding observed can partly be explained by gender (Mehio-Sibai et al., 2005), as female workers compared to male workers were more likely to report effort-reward imbalance (and higher scores on effort and overcommitment, and lower scores on reward) and less likely to report alcohol-related problems. This is contrary to a recent German study which found that men reported somewhat higher effort and higher ERI while no differences were found for reward and over-commitment (Wege et al., 2018). In our sample, female workers were also more likely to have higher education and being somewhat younger. Furthermore, higher education was associated with lower risk of alcohol-related problems and younger age was associated with higher risk of alcohol-related problems in general. The patterns observed between the different ERI components and age and educational levels are similar to results from other studies although inconsistencies across study populations have also been reported (Siegrist et al., 2004).

### Effort-Reward Imbalance and Alcohol

It has been suggested that exploration of job-strain as formulated by Karasek et al. (1998) is particularly relevant in industrial work samples, while studying the individuals’ responses to their work in terms of efforts and rewards may be more appropriate for tertiary sector employees (Tausig and Fenwick, 2011). ERI focus on the work role in a social perspective, as well as the jobholder’s individual coping pattern and need for control (Stanhope, 2017). It also includes aspects such as salaries, promotion prospects, and job stability which directly links stressful work-related experiences with broader labor market conditions (Peter et al., 2002). The relevance of broad labor market conditions on workers’ mental health are evident from studies investigating potential impact of economic crisis on for instance perceived stress, anxiety, depression (Giorgi et al., 2015; Mucci et al., 2016). In relation to alcohol, there is evidence that consumption levels, alcohol-related problems, and excessive mortality due to alcohol consumption increases during an economic crisis or job instability (de Goeij et al., 2015; Mucci et al., 2016; Frone, 2018). Arguably, the effort-reward imbalance framework is a better gage for stressors experienced in the service-economy which dominates Western countries (Muntaner and O’Campo, 1993; Muntaner and Schoenbach, 1994). Based on these considerations we expected that high effort, low reward, high effort-reward imbalance in favor of effort and high levels of overcommitment would all be associated with alcohol-related problems. Our findings support this for all aspects of the model, although reward was not significantly associated alcohol-related problems after adjusting for age, gender and education. This is in contrast with some previous findings (Kouvonen et al., 2005; Guo et al., 2006), but consistent with one study employing a compound measure of alcohol-related problems (Head et al., 2004).

Although most studies with a focus on alcohol-related factors and stressful working environment have used alcohol consumption *per se* as outcome measure, a few have included compound measures of both consumption and potential negative consequences of the consumption (Head et al., 2004; Guo et al., 2006; Fear et al., 2009). But even in studies which use the same alcohol measure as us (AUDIT), the findings are mixed (Guo et al., 2006; Fear et al., 2009). The discrepancy between our study and previous findings may be due to differences in sample size, measurement instruments, statistical modeling, response rates – but might also reflect true differences in the association between stressful working environment and alcohol-related problems in different populations.

In summary our study investigate a theoretical concept of stressful work that captures features of modern, post-industrial work and employment and find a consistent association between effort-reward imbalance and alcohol-related problems. Distinct from previous reports on the topic, the present study emphasizes alcohol-related behavioral consequences rather than being restricted to amount of alcohol consumption, thereby illustrating public health implications of our findings. Also, by exploring the explanatory potential of the ERI model beyond the usual summary analyses, separate and combined effects of the ERI components are demonstrated which have not been presented in previous studies.

### Implications

Our findings suggest that the aspects of a stressful working environment as highlighted by the effort-reward imbalance model, is relevant for our understanding of alcohol-related problems. From a public health perspective, this suggests that a combination of individual and work-related factors should be taken into account when assessing the likelihood of alcohol-related problems. At the same time, it has been underscored that the potency of work stress as risk factor for substance use is limited (Landsbergis et al., 1998). Alcohol use is determined by several distal and proximal factors, both individual and contextual, in a complex matrix. Factors related to work are merely a small part of this. It is, however, evident from this study that high levels of effort/reward-imbalance in combination with high levels of overcommitment seems to confer risk at a level of public health relevance.

### Strengths and Limitations

**FIGURE 4:**
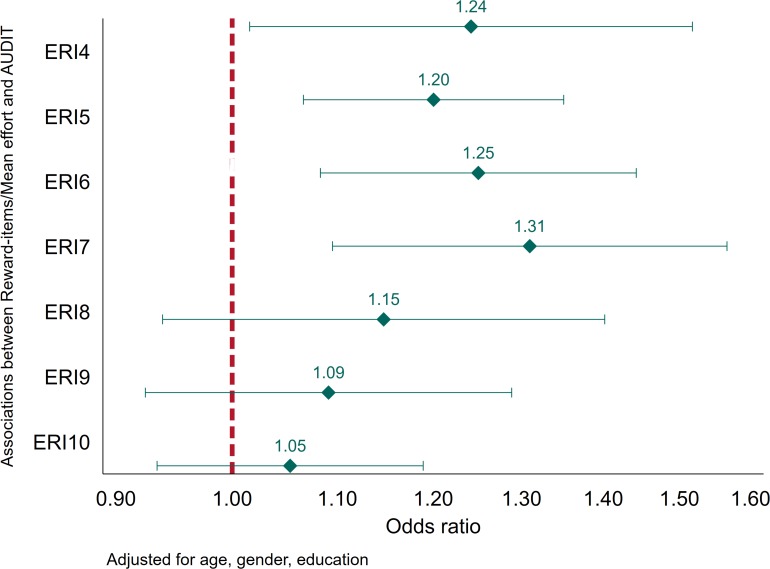
Associations between the ratio of individual Reward-items and mean Effort, and alcohol-related problems (AUDIT ≥ 8). Estimates adjusted for age, gender, and education (*N* = 4,314).

The present study have several strengths. First, it is based on recently collected information from participants from several different companies in Norway. Second, the study included a widely used measure of both alcohol consumption patterns and potential consequences of this consumption. The present study also have some limitations that should be borne in mind when interpreting the reported findings. First, although the reliability was good for most of the sub-scales, the reward-scale had a rather low reliability. This may have biased our findings, most likely by diluting the reported associations based on this sub-scale. We did, however, perform a *post hoc* sensitivity analysis of associations between the ratio of individual Reward-items and mean Effort, and alcohol-related problems (Figure 4). In the *post hoc* analyses, all of the point-estimates indicated higher odds for alcohol-related problems, and four of the seven ratio-items were statistically significant. Second, the proportion of missing information on AUDIT was substantial in the present study, and associated with sociodemographics. Missing on information about alcohol may be due to respondents being abstainers and considering AUDIT irrelevant. It is also likely that participants were more reluctant to answer questions regarding alcohol since the study was done in collaboration with the company they worked for. The latter may be particularly relevant among high-level consumers, or participants who consider themselves to be high-level consumers. We chose to handle missing data by using multiple imputation, assuming that missing data was at least missing at random (MAR). This implies that the probability that for instance AUDIT-scores is recorded at an individual level depends on some other factors we have recorded, for instance education, and that this probability is fairly constant within specific levels of the other recorded factors. If this is not the case, missing is considered missing not at random (MNAR), and multiple imputation will not remedy the potential bias induced by the missing data (Sterne et al., 2009). Whether or not this was the case in this study is by definition not possible to assess, and we cannot rule out that we were not able to compensate for any bias introduced by the mechanisms behind our missing data. Third, unlike some of the previous studies, our study is cross-sectional and we are not able to determine any potential causal pathways. As such, we cannot rule out reverse causality. For instance, participants experiencing alcohol-related problems may face or perceive more work-related problems, thus affecting their answers to the ERI-questionnaire. It may also be that the reported associations would be different if the study was longitudinal, as was reported by Heikkila et al. (2013). Lastly, the data is based on participants from Norwegian companies, and the findings may not be generalizable to other contexts with substantially different working cultures and working regulations.

## Conclusion

Our findings suggest that effort-reward imbalance and overcommitment is associated with increased likelihood of alcohol-related problems. These findings indicate that individual and work-related factors should be taken into account collectively when aiming to determine the impact of psychosocial work environment on alcohol-related problems. Due to the cross-sectional nature of the present study, we are not able to determine the direction of the associations, and future studies should aim to investigate this.

## Data Availability

The datasets generated for this study will not be made publicly available due to data protection restrictions (GDPR), restrictions in Norwegian Law and ethical considerations. Data from the WIRUS study is available for Research purposes by application to the WIRUS Project Group provided national and international guidelines for research is followed.

## Ethics Statement

The studies involving human participants were reviewed and approved by the Regional Committee for Medical and Healthcare Research in Norway (no. 2014/647) appointed by the Norwegian Ministry of Education and Research. The patients/participants provided their written informed consent to participate in this study.

## Author Contributions

RA is the principal investigator and project manager of the WIRUS-project, designed and piloted the WIRUS-screening study, and took part in planning and monitoring for recruitment of companies. RA and MT collected the data. JS carried out the initial literature review for the “Introduction” and “Discussion” sections, performed the initial data analysis, and wrote the first draft of the manuscript. MT, TB, JV, BS, and RA involved in the preparation and planning of the statistical analyses, and reviewed and contributed to all parts of the written manuscript, including suggestions to revision of statistical analysis, changes to manuscript content, suggesting further research literature, as well as the interpretation of the findings. All authors made critical revisions and provided intellectual content to the manuscript, approved the final version to be published, and agreed to be accountable for all aspects of the work.

## Conflict of Interest Statement

The authors declare that the research was conducted in the absence of any commercial or financial relationships that could be construed as a potential conflict of interest.
